# Assessing the Impact of a Leadership Development Programme for Community Pharmacy Neighbourhood Leads in South East London

**DOI:** 10.3390/pharmacy12060164

**Published:** 2024-11-02

**Authors:** Finlay Royle, Sarah Guard, Ricarda Micallef

**Affiliations:** 1 NHS South East London Integrated Care Board, 160 Tooley Street, London SE1 2TZ, UK; finlay.royle@selondonics.nhs.uk (F.R.); sarah.guard@selondonics.nhs.uk (S.G.); 2 Department of Pharmacy, Penrhyn Road Campus, Kingston University London, London KT1 2EE, UK

**Keywords:** community pharmacy, pharmacist, primary care, leadership, training, development, communication, pharmacy first, integration, transformation

## Abstract

Community pharmacists continue to deliver a growing number of services, with an increased need for collaborative work between local teams in community settings. In South East London, the Integrated Care Board and Pharmacy Alliance have invested in the development of community pharmacy neighbourhood leads (CPNLs), who represent community pharmacies in a locality and provided a tailored five-session leadership course that ran between November 2023 and March 2024, covering theory and implementation. This study aimed to evaluate the experiences of the CPNLs and other colleagues who participated in the leadership development programme. Participants were asked to complete an evaluation survey after each session, along with individual interviews taking place with those who volunteered. Ethical approval was received. There was a total of 37 participants at the sessions, and 7 participated in an interview. Overall confidence increased throughout the course, with males statistically more confident than females. Relationships with new stakeholders also increased throughout the course. The proposed actions after each session echoed the topics covered, with planning meetings being prioritised. The interviews highlighted new opportunities identified and being acted on, a greater understanding of the role, and an increase in confidence and key relationships. Barriers in the role included time for meetings. Overall, investment in the CPNL role showed that learning from the programme was applied in practice, with an increase in confidence and understanding of the role and improved local relationships. The findings from this study can be used by others to support community pharmacy transformation and integration.

## 1. Introduction

Community pharmacy integration with other primary care NHS service providers, such as general practice, is a key NHS strategy for the transformation of health services, and the sector is well placed to deliver the ambitions of the strategic approaches detailed in the Fuller stocktake report [1] and the NHS Long Term Plan [2]. Through the utilisation of its skilled workforce, community pharmacy can progress Integrated Care System (ICS) system objectives to deliver patient-centred care, improve patient outcomes, and reduce health inequalities whilst deepening its role at the system level. Pharmacists are uniquely placed at the intersection of many patient-facing disciplines to support synergistic care [3].

In 2019, the Department of Health and Social Care (DHSC), NHS England, together with the Pharmaceutical Services Negotiating Committee (PSNC) (Community Pharmacy England since June 2023), agreed to the NHS Community Pharmacy Contractual Framework (CPCF) for England [4]. The framework describes the vision for the transformative role that community pharmacies will have, particularly supporting the prevention, identification, and management of long-term conditions, alongside a 5-year investment of GBP 13 million between 2019 and 2024 [2,4,5]. The potential for community pharmacy to increase capacity and expand access to key health services was demonstrated through its reaction to the COVID-19 pandemic [6], and a shift to a more clinically focussed, outcome-orientated service model now sees pharmacies delivering a wide range of services, including vaccinations, health and wellbeing, physical and mental health screening, self-care, and medicines adherence support. Through the introduction of the national Pharmacy First service in January 2024, community pharmacy will continue to offer convenient access to safe and high-quality healthcare for patients with seven minor illnesses, following clearly described clinical pathways [7]. At an opportunity cost of GBP 56 per General Practitioner (GP) appointment saved [8], this frees up significant capacity in general practice, and there is further value to be gained by the healthcare system through increasing use of community pharmacy capacity and skills.

With the delivery of a growing number of clinical services by community pharmacies, there is a need for improved collaborative working between local teams in neighbourhood settings—specifically between general practice and community pharmacy—to maximise opportunity. In South East London ICS, community pharmacy integration has been identified as a key strategic priority. Furthering the concept originally identified through CPCF 2019, community pharmacy leadership at the neighbourhood level is vital to sustaining an approach for improving the quality and consistency of pharmacy services and consolidating local relationships [9]. To support this aim, the Integrated Care Board (ICB) worked with the South East London Local Pharmaceutical Committee (LPC) to support the establishment of the South East London Pharmacy Alliance, a body which acts on behalf of pharmacy contractors in the area and can compete for and deliver services across an entire population. Pharmacy contractors include independent pharmacies owned by the pharmacist and multiple pharmacies owned by corporate organisations. The Alliance has a key objective of building and maintaining leadership capacity amongst the community pharmacy network to support leadership succession planning and add depth to existing leadership capacity.

In 2022, Lambeth borough invested in funding community pharmacy neighbourhood leads (CPNLs), covering areas similar to Primary Care Networks (PCNs) [9], groups of GP practices which cover a population of between 30 and 50 thousand. The key responsibilities of the CPNL role are to provide leadership and representation for the community pharmacies in the neighbourhood, to develop close working relationships with PCN clinical directors with common goals identified, and thus to support communication, awareness, and implementation of pharmacy services locally. CPNLs are community pharmacists who have put themselves forward and been assessed and selected by the Pharmacy Alliance as having the skills to meet the role description. They are supported by the ICB medicines optimisation team and the Alliance, and at the time of the Lambeth investment, they were funded four hours monthly [9]. The leads started in October 2022, and an informal development programme was established to support them in their role. An evaluation of this programme [9] was conducted to evaluate their experience in the role, identify barriers to communication along with good practice, and identify their learning and development needs with a view to shaping future improvements to the CPNL programme as it expanded across South East London (SEL). This study comprised semi-structured one-to-one interviews with the Lambeth CPNLs, resulting in qualitative data on their perceptions and experiences in the role that were subsequently analysed [9]. The interviews explored support requirements for the CPNLs and identified that the leads needed further leadership training and development to improve their interprofessional communication skills, ability to influence, and confidence in engaging and building relationships with stakeholders.

In March 2023, through partnership with the LPC, the SEL Workforce and Development Hub, and the Alliance, the ICB medicines optimisation team invested in 24 CPNLs, funded for 8 h a month and working across all six SEL boroughs, thus developing the CPNL role into a system-wide integrated project. Funding was also identified for a comprehensive leadership development programme to support the 24 CPNLs. Building on the findings of the previous evaluation, the ICB medicines optimisation team worked with an identified training provider, Kaleidoscope Health and Care [10], the LPC, Alliance, and the established Lambeth CPNLs to co-design a leadership development programme that aligned to the ICB’s ‘five characteristics of an effective system leader’ [11] and also to wider leadership programmes delivered by Kaleidoscope Health and Care in SEL. The findings of the previous evaluation were reflected in the three core themes of the leadership development programme, as agreed by the stakeholders during the co-design process—(1) building trust across boundaries, (2) driving purposeful collaboration, and (3) catalysing and embedding innovation. These also link to the Royal Pharmaceutical Society’s leadership development framework, which includes recommendations relating to inspiring shared purpose, leading with care, sharing the vision, and influencing for results [12].

The SEL CPNL leadership development programme was delivered across five monthly sessions between November 2023 and March 2024, one a month, and all 24 recruited CPNLs were invited to participate alongside members of the LPC, Alliance, and ICB medicines optimisation team. The first and last sessions of the programme were delivered face-to-face, with the remaining sessions conducted virtually. Each session covered both theory and practical implementation based on the identified core themes. Information about each session’s content can be found in Table 1.

This study aimed to evaluate the experience of the CPNLs and other colleagues who participated in the leadership development programme. This included their confidence in the role, relationships, and actions completed carrying out the role.

## 2. Materials and Methods

### 2.1. Study Design

A mixed methods inductive study was completed using both post-session surveys and interviews. Questions were created to gain insight into current roles, capture the learnings from the programme, and determine how these were used in practice. Participants were asked to complete post-session surveys to explore their thoughts about and experiences of the leadership training. The survey after session one comprised 17 questions, sessions two–four were identical with 23 questions, and post-session five asked 32 questions. Five different survey links were created on Microsoft Forms. The evaluation forms were broken into five sections covering demographics, confidence in the role, current relationships, session evaluation, and actions taken/planned. A 5-point Likert scale was used for rating questions, with actions taken and planned being free-text.

Session one asked about motivation to participate in the programme, which was not included in future surveys. Sessions two to five asked whether there had been previous attendance, along with free-text responses to explain the scores given. The survey post-session five asked more questions about the overall experience of the programme.

Qualitative data from community pharmacy neighbourhood leads were obtained using semi-structured, one-to-one interviews to gain insights into individual practice. An interview proforma comprising 12 questions was designed. It aimed to understand the CPNL role, experiences of the leadership programme, and how practice has changed because of attendance and ongoing support needs.

The interview schedule received content face validation from the ICB medicines optimisation leads. They were not included in the interviews. The interview schedule can be found in Appendix A. The COREQ checklist was used to ensure that the design and analysis were robust. This can be found in Appendix B.

### 2.2. Participants: Sampling and Recruitment

Human participants were involved in data collection through survey completion and semi-structured interviews.

All the participants in the leadership programme, including CPNLs, ICB, and LPC colleagues (all of whom are pharmacists), were invited to complete an online survey at the end of each session by the programme facilitator. The purpose of the survey was explained, and participation was voluntary. Whilst planning each session, time was allocated within the agenda to ensure evaluation was integral to the experience. An online Microsoft Forms link was provided to the participants so they could complete the survey using their laptops or smart devices. Paper copies of the survey were available if required.

Potential participants for the interview were identified through a question at the end of survey five asking whether they would be willing to take part, along with an email to those who had attended previous sessions but not session five. They were contacted through the ICB leads, with the participants’ email addresses being provided to the researcher, who had no prior relationship with the participants.

### 2.3. Data Collection

Survey data were collected at the end of each learning session. During session one, all participants were given a paper copy of the participant information sheet informing the participants about the aim of the project and how their responses would be used. All the participants in any session were also emailed a copy prior to the session. If the participants wanted more information, they could ask questions at all sessions before completing the survey. Implied consent was given on completion of the survey by the participants, and they were advised that they were under no obligation to take part. They could withdraw from this study up to the point of final submission.

One-to-one interviews were conducted with all who agreed to participate. The interviews were held over Microsoft Teams arranged at a mutually acceptable time, according to the availability of the pharmacist participants. The participants who agreed to participate in an interview were emailed an information sheet, again outlining the study aims and objectives and the background of the researchers, including the right to withdraw. They were also sent a consent form to read, sign, and return prior to the start of the interview. Although written consent to record the interviews was provided by each participant via email, verbal consent was also requested prior to each interview, ensuring the participants agreed to be voice-recorded. All interviews were transcribed verbatim from the audio recording prior to deletion. No other notes were made during the interviews. The interviews took place during May 2024. In order for a range of views and experiences to be recorded, all the participants who agreed to be interviewed took part. One member of the research team (RM), PhD, with qualitative research experience, provided academic leadership and oversight for all the interviews and transcriptions. The surveys and interviews were conducted by a member of the Kaleidoscope research team, solely completing this task to ensure no previous relationship or bias. Kaleidoscope were commissioned to provide this as part of their service offering. Only the Kaleidoscope researcher and the participant were present during the interviews.

All interview recordings were stored locally on a password-protected online platform and were instantly deleted after transcription into Microsoft Word.

### 2.4. Data Analysis

The survey responses from each of the five surveys were downloaded from Microsoft Forms into Microsoft Excel. Descriptive statistics, including percentages, frequencies, and weighted means, were used. As the data were non-normally distributed and ordinal in nature, Mann–Whitney U tests were used to detect any associations between responses. Statistical significance was assumed where *p* ≤ 0.05. A *p*-value ≤ 0.05 was considered to be statistically significant. Free-text responses were analysed through thematic analysis.

The interview responses were analysed using content analysis led by the themes and subthemes emerging from the interview questions to allow responses to be compared between participants. This was completed by one member of the research team (RM) with another male member of the team (FR), MSc, reviewing all transcripts for accuracy. A manual analysis was completed. The transcripts were reviewed for any transcription errors and then to enable immersion. Upon review, each participant was emailed a copy of their interview transcription to confirm its accuracy.

Direct quotations are used in the results to illustrate the findings.

### 2.5. Ethics

This study received ethical approval (3396) from the delegated ethical approval team at the relevant institution.

## 3. Results

### 3.1. Participants

Over the five sessions, there was a total of 37 participants. Sessions one and five were face-to-face, and the others were online.

Of the 37 participants, 16 attended four or five sessions. Ten participants only attended one session. Full demographic details can be found in Table 2.

The number of sessions attended is as follows:

- Attended one session—10.
- Attended two sessions—four.
- Attended three sessions—seven.
- Attended four sessions—11.
- Attended five sessions—five.

The attendance for the sessions is listed below. For the 16 who attended four or five sessions, the values are in brackets.

- Session 1—27 (13).
- Session 2—22 (14).
- Session 3—19 (16).
- Session 4—12 (11).
- Session 5—28 (15).

### 3.2. Survey Responses

The motivation for joining the course was captured by free-text responses. From the 34 responses received, the themes included wanting to improve their skills, collaborations, and local networks. The link to improved patient outcomes and impact on health was also seen.

Overall confidence in the role increased during the course of the programme (Table 3), with those attending four or five sessions showing increased confidence over those who attended three or fewer sessions. However, the difference in confidence scores for those attending more sessions was not significant (*p* = 0.17068). Looking at gender, however, males were statistically more confident than females across the programme (*p* = 0.02852). The number of participants who had been practising for 20 years or more, compared with those with ten or less years of practice, was significant (*p* = 0.0139).

Comments regarding the confidence score for session 5 showed that the participants were more confident in their skills, had a better understanding of the role, and were also eager to keep learning.

Relationships with key stakeholders also increased during the course of the programme (Table 4), with those attending four or five sessions rating their relationships as stronger than those who attended three or fewer sessions. However, the difference in relationship scores was not significant for any group (community pharmacists—*p* = 0.77948; GPs—*p* = 0.33706, ICB colleagues—*p* = 0.56192; other clinical leaders—*p* = 0.22628). There was also no statistical difference in any of the relationship scores by gender or length of practice. When looking at those who attended four or five sessions to see a clear journey, between session 1 and session 5, relationships with the ICB medicines team saw the biggest increase (+1.3), with other community pharmacists seeing the lowest increase (+0.53).

In terms of usefulness (Table 5), there was limited difference across the number of sessions attended (*p* = 0.52218), by gender (*p* = 0.68916), or length of practice (*p* = 0.58232).

Key learnings post-session emphasised the topics that had been covered. Planned actions were similar after each of the sessions, with planning meetings with local stakeholders coming up as the most prioritised action. Continuing to build networks and local collaborations was also highly mentioned.

### 3.3. Interviews

#### 3.3.1. Demographics of Interviewees

There were seven participants in the interviews, of which four were contractors (and CPNLs), two were pharmacy managers (CPNLs), and one was a senior leader from an SEL community pharmacy representative organisation. Five of the interviewees were male and two were female. Three interview participants had over 20 years of experience practising as a pharmacist, three participants had between 11 and 20 years, and one participant had between 6 and 10 years. The interviews lasted between 24 and 36 min.

#### 3.3.2. Understanding More About the Leadership Programme

When asked what leadership skills individuals hoped to gain or develop as part of the programme, the answers were varied and included building on current skills (*n* = 2), building relationships/networks (*n* = 4), communication skills (*n* = 2), confidence (*n* = 2), and being able to learn from each other (*n* = 2), along with overcoming any barriers (*n* = 1).

Having new opportunities was also highlighted.

*I mean, I’m just really grateful for the programme, you know, just for some of us who are clueless, as I say, I spend most of my time shackled behind the dispensary so these roles that they’ve put on us are… it’s a look into the unknown for a lot of us.*

Participant 7.

When asked how the programme helped the individuals in their roles, overall, the programme has enhanced leadership, communication, and confidence, fostering integration and progress within the neighbourhoods. With leadership and integration, it has brought together voices, broken down barriers, and integrated community pharmacy more efficiently.

Four of the seven participants noted that community pharmacy had previously been outside of local health and care organisations, but the programme has supported integration.

*I think before, community pharmacy always felt separate but now with this programme with all the support we are getting, it is definitely feels more integrated with things locally.*

Participant 2.

The participants grew in confidence (*n* = 3) and influence with increased communication with GPs and other local partners (*n* = 3), as also seen in the survey results. Communication was enhanced, with six out of the seven interviewed saying that they have been adapting their approach according to different people’s needs and styles and knowing that a one-size-fits-all approach to communication will not bring the most effective outcomes.

*I have to sort of change and adapt the style to suit the needs of the person that I am speaking to.*

Participant 4

*Yes, it has helped because you’re able to use a different dialogue, a different way of influencing the primary care network, clinical directors, the GPs, the practice managers.*

Participant 6.

*I have gained a good level of confidence and not just understanding my role, but also how to engage with different stakeholders.*

Participant 7.

For one individual, learning the theories to underpin knowledge re-enforced their current practice and backed up behaviours with academic rigour.

*[The course] provided me with assurance about my own capability and translated my knowledge into academic rigor… So it was being able to see that theory and to understand some of the language and the way it was expressed.*

Participant 3.

Wanting to overcome barriers was noted by one candidate for the reason they registered for the programme, and two noted that the programme helped them overcome previous issues, with the guest speakers giving useful tips and sharing experiences.

*Some of the guest speakers… this was very inspiring and very motivating for me, because... everybody has to overcome obstacles and problems and it was inspiring to understand how other people were experiencing the same problem.*

Participant 5.

Building relationships locally was a success, with individuals learning from each other and supporting each other.

*We are developing leaders and actually we can see that we’re working as one community pharmacy voice now.*

Participant 1.

*I really appreciated that ability to connect and learn from other pharmacists in the programme. It was really significant.*

Participant 7.

*I do expect these community pharmacy neighbourhood leads to become integral members of the integrated neighbourhood teams.*

Participant 6.

Participant 5 suggested that in order to further strengthen relationships, more stakeholders could be included in future programmes.

*So maybe the next time, maybe to involve... not only community pharmacy, but maybe to involve more GP practice as well in the programme so we can learn together and know more about each other and then work together better.*

Face-to-face interaction with colleagues was also noted as having benefits, with five of the seven saying they had arranged face-to-face meetings with other local leaders—pharmacists, PCN directors, and GPs.

*[community pharmacy leads] actually put themselves in situations they wouldn’t have done normally to go outside their comfort zone and experience having face-to-face meeting with a PCN or a face-to-face meeting with a PCN clinical director.*

Participant 1.

*I went to have a coffee with one of the pharmacists, a practice pharmacist and you know, we always communicate by email or we will speak in a rush on the phone. It makes such a big difference even to stay only half an hour and to look at each other and to stay away from our own environment full of distraction and now the communication has become much, much stronger.*

Participant 5.

*I message them or I send them information but I also go and see them just to be able to get a bit of a bit of just face-to-face, that just helps people to feel a bit more reassured that they do know you and know you are there to help.*

Participant 3.

When talking about the structure of the programme, face-to-face meetings were mentioned as being extremely beneficial for networking; however, it was noted that having online meetings was also beneficial for evening sessions to allow participation.

*From a point of view of practicality, doing a Teams event in the evening where people can make it, it makes a lot more sense. But having that face-to-face interaction and being able to talk to each other and doing the group bits and having a lot more interaction was I think it became a lot more beneficial.*

Participant 1.

*Making sure there is an opportunity for virtual and face-to-face because I think it’s really important that virtual is really useful but face-to-face is really important.*

Participant 4

*I preferred meeting face-to-face, because it is more engaging. You got more opportunity to interact and to practice as well.*

Participant 5.

*But I think what could be helpful is more face-to-face time. If we did more leadership programmes then like I think if you go to a sort of retreat for like 2–3 days because you will move away from your environment as well from the from distraction, maybe it will become more effective.*

Participant 7.

#### 3.3.3. Understanding the Role

When asked about main stakeholder groups, all seven mentioned GP surgeries and their local community pharmacies, four mentioned PCN clinical directors, three mentioned the ICB, two mentioned the Pharmacy Alliance, and one mentioned the LPC. Two mentioned the other neighbourhood leads. Collaboration with stakeholders was mentioned as key to successful outcomes.

*I engage with the clinical directors within the GP practices…. Obviously the rest of the neighbourhood leads and because as peer group it’s quite useful to be able to share information and then also anybody else you that you bump into in doing the job, you’re looking to build relationships.*

Participant 3.

*In terms of stakeholders, the main one is obviously the other community pharmacists within the neighbourhood.*

Participant 4.

*There’s more collaboration because if we succeed, we will succeed together we will not be able to succeed only on an individual basis. Then another stakeholder is the surgery, we’re dealing with quite a lot of surgery…The Clinical Director of the PCN too, because they are the one that opened the door to each individual surgery.*

Participant 5.

The meetings that were attended were different for each individual, dependent on their current roles, with meetings including borough meetings, the pharmacy Alliance, and LPC. Two individuals also mentioned the monthly neighbourhood lead meetings.

When asked how trust was being built with key stakeholders, all seven mentioned that increased two-way communication was the key to success. Reciprocal understanding of roles and how to support each other was essential.

*In terms of building relationships and trust, with the pharmacist, it’s testing times and having that person and knowing that you can trust that relationship you’ve built there and actually you’ve got someone to go to makes a difference.*

Participant 1.

*I suppose it’s that bit about understanding what their wants are, what their needs are, uh, understanding their challenges and concerns and then thinking about how what you do to address those.*

Participant 3.

*I went to the surgery and I took the time to understand how they’re working, how they’re operating in the surgery… it was understanding the problem, and then working together to make it better, and adjust it accordingly to their needs as well.*

Participant 5.

To support the role, having a WhatsApp group of other leads was useful, facilitated by the Pharmacy Alliance, along with the monthly neighbourhood lead meetings. Going forward, more close work with the medicines optimisation team was requested, along with support to build channels of communication to all surgeries, where key contacts keep changing.

*But what has been good is because we set up through the Pharmacy Alliance, the WhatsApp group for the leads and we’ve got an email link between them all.*

Participant 1.

*I think that the support has been great I don’t think they could have done much more. I’ve got everyone’s numbers so I can speak to them whenever I want, right? So that’s a key thing, there’s no barriers.*

Participant 2.

*So what’s been really helpful is the relationship with the Meds Op team initially to help to broker some of those contacts and those first meetings and being able to broker relationship.*

Participant 3.

*I think in terms of the support what would be really helpful, would be opening up the channels of conversation with those practices that have not necessarily had someone be a key contact to engage with.*

Participant 4.

*So it would be nice to be a bit more involved with the Meds Optimisation team, I’ve made inquiries about that.*

Participant 7.

#### 3.3.4. The Role and the Leadership Programme

In terms of challenges or barriers, six out of the seven mentioned the time needed to attend meetings or invest in the relationships needed. Changing habits and continued communication was also noted. However, the programme provided the skills to feel more confident and reach out to stakeholders to have appropriate conversations.

*I think my challenge I have already outlined really in terms of admin and time with organising and I’m not sure the programme could’ve done anything to support that, it was more about skills to help relationships and things like that.*

Participant 2.

*So the first stage was being able to get to know who they were, what they did, what their own thoughts were, which goes back to that bit about what do they see as their challenges, opportunities, what can we do to address those and at the same time.*

Participant 3.

*I think the challenge that I’ve felt is to do with having to take time out of the business… Having that training, giving you a way of looking at how you communicate with people, how you understand people’s behaviours, how they are, so that you can adapt your style to them as well has been very useful.*

Participant 4.

*I think the programme absolutely has helped; it was that sort of reframing of the attitude.*

Participant 6.

*I think the programme has given me a good starting point to be able to, as I say, start making these engagements and communications. I feel like the rest of it is up to us.*

Participant 7.

To sustain and build upon the knowledge and skills gained from the leadership development programme, five of the seven suggested they would revisit the resources that were provided, to keep reminding themselves of the key messages, whilst reflecting on what they learnt. All seven mentioned that they wanted to maintain the relationships formed through the programme, along with forming new relationships.

*The biggest thing that’s come out of it has been the network that’s been established… I think really, it’s just about continuing that communication and kind of building on what we have and more of that support and that network rather than trying to establish anything further right now.*

Participant 1.

*I suppose part of it is becoming part of the leadership programme system wide, I still know most of the people, most people probably still know me, but actually re-establishing some of those relationships at that senior level across the system and getting to know who the new leaders are that I’ve not met.*

Participant 3.

*So I would like to stay in touch with all my colleagues and to keep learning altogether because everything is still quite new and because there’s still a lot of a lot of problems that we need to face together.*

Participant 5.

*For me, it’s a continual process. It’s not something that just going to be put away on the computer file and never to be seen again.*

Participant 6.

Increased visibility and participation in local meetings were also highlighted by one participant.

*Ongoing, I think it’s making sure I attend more meetings… I think that’s sort of like my personal aim for the next year, to try and advocate more for community pharmacy locally doing more presentations and things.*

Participant 2.

## 4. Discussion

### 4.1. The Programme

The learning from our initial pilot in Lambeth [9] and the first year of this SEL-wide programme has informed the development of our community pharmacy leadership programme. Leadership supports pharmacists to be proactive in their roles, regardless of their position [14]. The programme aligned to the ICB’s ‘five characteristics of a South East London leader’ [11] and drew out key priorities for the CPNL cohort through the co-design process.

The programme was designed to be flexible enough to enable busy healthcare professionals to participate, acknowledging the constraints of time for CPNL activity (8 h per month), and therefore involved some meetings that were online and in the evening. Whilst the participants reflected that this was helpful in balancing priorities and enabling attendance, they also reflected that more face-to-face development would have enhanced their learning and in establishing their network. Whilst a preference was seen for more face-to-face time as part of the programme—as seen in a previous study on pharmacist continuing professional development preferences [15]—this was not reflected in their approach to the role. This echoes a previous paper indicating that virtual interactions do not negatively affect peer perceptions [16]. Our participants considered face-to-face interactions with stakeholders to be personally important to them, as reflected in comments relating to the benefits of meeting individuals in GP surgeries or in social environments.

Because of the lack of being physically together, virtual settings require more task focus and relationship-orientated leadership energy to achieve the same outcomes [17]. However, the participants generally reflected that they came out of the programme with a clear sense of purpose, developed and even tested new approaches for engaging with neighbouring practices, and improved their understanding of how to frame discussions with their peers and stakeholders. The increase in CPNLs’ confidence is reflected in how they engaged with stakeholders, and overall, the programme opened a window for CPNLs to explore new ways of working, reframing how they engage with stakeholders, in particular, GPs and PCN Clinical Directors. The increase in confidence scoring is corroborated by the participants’ comments.

### 4.2. Demographics

Our cohort was experienced, with 26 of 37 participants in practice for 11 years or more. The cohort was also over-represented by men, but otherwise, it was broadly representative of the pharmacy profession as a whole and reflected the make-up of our local population. Across SEL, the pharmacist workforce is overwhelmingly female (78.5%) [18], and so, in line with the Joint National Plan for Inclusive Pharmacy Practice in England [19], consideration will need to be made as to how female pharmacists can be better supported and encouraged to apply for leadership roles. This is being addressed through the development of the SEL ICB pharmacy workforce strategy.

### 4.3. Increased Relationships and Skills

As seen in a previous study and echoed here, leadership education gives confidence to pharmacists to support scaling of clinical services and ultimately support patient care [20]. The results showed a statistical difference in the confidence of males over females, echoing previous work [21,22], and those with over 20 years’ experience were more confident than those with less than ten years’ experience. It is important to recognise that leadership change takes time [23], and this programme continues to work towards transformational change for the profession and patients. Through ongoing work, those with less experience will continue to build confidence, as small learnings can change confidence, and individual self-confidence helps to determine successes [24]. During COVID-19, it was seen that being female and younger was associated with lower self-confidence in community pharmacists [25]; therefore, it is important to continue to give confidence to newer professionals and remove barriers to participation.

Whilst not statistically significant, the baseline and improved levels of confidence varied according to specific groups. For a group of pharmacy professionals who are starting in a newly established leadership role, and working in relatively isolated environments, it was unsurprising that confidence was greater when considering relationships with other groups of pharmacy professionals, and less so when considering other clinical leaders in the wider health and care system. Supporting leadership development alongside other clinical leaders within the ICS is a future priority for the CPNL programme.

The programme also resulted in increased communication with relevant stakeholders within neighbourhoods. This communication is essential to build trust and meaningful collaborations [26], echoing the aims of the programme. Continued and sustained communication will increase role satisfaction and add value to professional contributions. Increased confidence and communication support the demonstration of the pharmacist’s unique skills and role [27].

The programme encouraged pharmacy leaders to work more closely with their nearest GPs and other healthcare professionals. This is crucial for sustaining high-quality care to allow for a continued understanding of each other’s roles [28,29] and to continue to build trust and collaboration in patient care [26]. It is known that frequent, informal communication enhances interprofessional collaborations [30].

### 4.4. Limitations

Our research was focussed on a small group of individuals, all with varying experience and backgrounds. So, whilst our findings are highly specific to this cohort, broader conclusions can still be drawn about how to plan, design, and organise similar leadership roles and development programmes elsewhere within healthcare systems.

These roles are new, and the clinical leadership programmes in South East London ICB (and predecessor organisations) have generally not included community pharmacy leadership roles. Therefore, building community pharmacy leadership capacity has taken time and has been challenging, especially for a cohort of healthcare professionals who are physically isolated and have limited interaction with healthcare leaders outside of their own immediate environment. This includes, in some cases, a limited understanding of or implementation of the role, struggling to engage GPs who see pharmacy as competitors, and competing priorities within their own pharmacy environment to create the capacity needed. By integrating the CPNLs into broader clinical leadership forums and development programmes, and the recent recruitment of a clinical lead for community pharmacy digital systems, we aim to further address these challenges.

Other factors impacting implementation included the launch—midway through our programme—of the national Pharmacy First service expansion [7]. This was implemented at pace and had a significant impact on participants’ capacity to participate. This was reflected through reduced attendance in sessions 2, 3, and 4, which were all online. Therefore, the competing pressure of implementing the Pharmacy First service may be a factor that reduced participation levels in the online sessions.

Our ability to measure the impact of the CPNL role on CPCS referrals was also limited by the implementation of the national Pharmacy First service expansion, as the number of services that can be referred to pharmacies increased significantly. GP-to-CP referral activity increased significantly because of the increased range of services, so assessing the relative impact of the CPNLs is hard to gauge. More broadly, the successful implementation of services such as Pharmacy First requires a broad strategic approach, with multiple levers needed to bring about change. The impact of the CPNL’s role as part of a broader range of measures should be considered in future evaluation.

The ICB underwent an organisational restructure during the implementation of this programme, resulting in new ways of working and changes to existing teams and personnel working to support community pharmacy integration. Following the ICB restructure, there is now an established ‘mini-team’ that supports community pharmacy programmes within the medicines optimisation team. This structure should ensure sustainable support to accelerate community pharmacy transformation and integration.

With these limitations in mind, we are confident that we can continue to develop and enhance the CPNL programme as further system integration continues.

## 5. Conclusions

Pharmacy leadership in South East London is evolving. Our cohort of CPNLs are diverse and have varying experience as pharmacists. They have clearly benefited from the leadership development programme but have some continuing needs as they develop in their role. Our research, conducted during the first 6 months into their role, demonstrates that the leadership development programme has been impactful, increasing the confidence of CPNLs in their role, helping them to better understand their role, and supporting or strengthening relationships with key stakeholders such as GPs across South East London. We are addressing the learning needs identified through this research to continue supporting the CPNLs with confidence, communication, networks, and relationships. This will inform the next phase of our programme.

## Figures and Tables

**Table 1:** SEL CPNL development programme session content.

Session Title	Session Objectives	Post-Session Learning in Action Task
Launch event (November 2023)	Connect with each other. Connect with the course requirements. Connect with the characteristics of a SEL system leader.	Observe a system or cross-organisational meeting
Building trust across boundaries (December 2023)	Develop a shared understanding of importance of cognitive and affective trust. Explore how to use cognitive and affective trust to develop and deepen credibility. Apply the principle of cognitive and affective trust to a real-time system-based challenge.	Reach out to a GP/community pharmacy/ICB colleague and ask them for coffee
Driving purposeful collaboration (January 2024)	Build an understanding of the reasons people collaborate. Introduction to concepts about how we (as collaborators) engage with the world around us and the approaches at our disposal to influence a positive outcome. Think about the practical steps that can be taken to improve collaboration in our areas.	Reflect on the social style of one person you collaborate with Think of one ask/offer for that person
Change that works (February 2024)	Build an understanding about change that works. Increase awareness of improvement methodologies. Explore the different types of innovation risks to consider in driving change.	Reflect on the idea of micro-actions Think of one micro-action for change in your practice and implement it, reflecting on the impact it had for you and colleagues
Look back, look now, look forward (March 2024)	Reflect on the individual and collective experience of the CPNL role. Gain insight into where we are at now against the five characteristics of an SEL system leader. Agree what we want to keep alive from this leadership development programme.	Networking event with SEL system leaders following the final session Meet and connect with other healthcare leaders across SEL through the Connect System Leadership Community [13]

**Table 2:** Demographics of the participants.

	All Participants (*n* = 37)	Participants Who Attended Four or Five Sessions (*n* = 16)
Role		
Contractor	21 (56.7%)	11 (68.8%)
LPC	1 (2.7%)	1 (6.2%)
ICB	4 (10.8%)	0 (0%)
Pharmacy manager	10 (27.0%)	3 (18.8%)
Pharmacist	1 (2.7%)	1 (6.2%)
Years in practice		
0–5	4 (10.8%)	1 (6.3%)
6–10	7 (19.0%)	2 (12.6%)
11–20	13 (35.1%)	5 (31.1%)
20+	13 (35.1%)	8 (50%)
Gender		
Female	11 (29.7%)	4 (25%)
Male	26 (70.3%)	12 (75%)
Ethnicity		
Any other Asian background	1 (2.7%)	1 (7.1%)
Asian/Asian British	8 (21.6%)	5 (35.7%)
Bangladeshi	1 (2.7%)	
Black/African/Caribbean/Black British	7 (18.9%)	1 (7.1%)
Chinese	1 (2.7%)	1 (7.1%)
Indian	11 (29.7%)	3 (21.4%)
Iranian	1 (2.7%)	
Irish	1 (2.7%)	1 (7.1%)
Pakistani	1 (2.7%)	
Prefer not to say	1 (2.7%)	
White—English/Welsh/Scottish/Northern Irish/British	4 (10.8%)	2 (14.3%)

**Table 3:** Confidence levels of participants (where 1 = no confidence and 5 = extremely confident).

	Session 1	Session 2	Session 3	Session 4	Session 5
**All participants**	3.7	3.77	4.05	4.17	4.14
**Participants who attended four or five sessions**	4	3.86	4.19	4.27	4.33

**Table 4:** Rated strength of participant relationships with key CPNL stakeholders over time (where 1 = no relationship and 5 = extremely strong relationship).

	Session 1	Session 2	Session 3	Session 4	Session 5
Community pharmacists in your neighbourhood					
All participants	2.96	3.18	3.53	3.33	3.37
Participants who attended four or fiv sessions	3	3.14	3.38	3.36	3.53
GPs in your neighbourhood					
All participants	2.7	3.18	3.39	3.42	3.29
Participants who attended four or five sessions	2.85	3.36	3.38	3.36	3.6
ICB medicines team in your borough					
All participants	2.74	3.18	3.89	4.33	3.89
Participants who attended four or five sessions	3.23	3.5	3.94	4.36	4.53
Other clinical leaders in your borough					
All participants	2.22	2.72	3.67	3.32	3.07
Participants who attended four or five sessions	2.31	3	3.31	3.63	3.33

**Table 5:** Overall usefulness of the sessions for participants (where 1 = not at all useful and 5 = extremely useful).

	Session 1	Session 2	Session 3	Session 4	Session 5
All participants	4.37	4.32	4.53	4.41	4.61
Participants who attended four or five sessions	4.46	4.14	4.5	4.45	4.4

## Data Availability

The data presented in this study are available upon request from the corresponding author. The data are not publicly available because of confidentiality issues related to the participants.

## Appendix A

Interview questions

- Please can you introduce yourself and describe your current role and the specific activities you do in relation to the community pharmacy neighbourhood lead role?
- Thinking back to when you signed up to the programme, what leadership skills were you hoping to gain or develop as part of the development programme? How would this add to any previous experience you have?
- In your opinion, has the leadership development programme helped you in your role as a community pharmacy neighbourhood lead?
- In your opinion, how could the leadership programme have better met your needs or expectations?
- In your current role, who are the main stakeholder groups you interact with regularly, and how would you describe your engagement with each of them?
- Can you describe the various meetings or forums you attend in your neighbourhood lead role?
- Reflecting on your communication approach, can you provide specific examples of how you have built trust and drive change with these stakeholders?
- Thinking about support you have received in your role, what has been helpful, and what else could have been helpful?
- What have been the most significant challenges or barriers you have faced in your role? Do you feel the leadership development programme has helped you address or overcome them?
- Looking ahead, how do you plan to sustain and build upon the knowledge and skills you gained from the leadership development programme in your ongoing work as a neighbourhood lead?
- Are there any other comments, observations, or suggestions you would like to share regarding the community pharmacy neighbourhood lead role or the leadership development programme?
- Is there anything else you’d like to add/discuss that we haven’t touched on yet?

## Appendix B. COREQ Checklist

	**Item No**	**Guide Guides/Description**	**On Page No**
		Domain 1: Research team and reflexivity	
Interviewer/facilitator	1	Which author/s conducted the interview or focus group? Kaleidoscope research team	Methods—6
Credentials	2	What were the reseachers credentials? RM—PhD, MPharm FR—MPharm, MSc	Methods—6
Occupation	3	What was their occupation at the time of the study? RM- Associate Professor FR—Associate Chief Pharmacist	Title page
Gender	4	Was the researcher male of female? Female of member of Kaleidoscope research team, RM—female, FR—male	Methods—5/6
Experience and training	5	What experience or training did the researcher have? RM—9 years of prior experience of qualitative research	Methods—6
		Relationship with participants	
Relationship established	6	Was a relationship established prior to the study commencement? No	Methods—5/6
Participant knowledge of the interviewer	7	What did the participants know about the researcher? E.g., personal goals, reasons for doing the research The Kaleidoscope research team were commissioned to conduct the interviews as part of their service offering. During session one, all participants were given a paper copy of the participant information sheet informing them of the aim of the project and how their responses would be used.	Methods—6
	**Item No**	**Guide Guides/Description**	**On Page No**
Interviewer characteristics	8	What characteristics were reported about the interviewer/facilitator? E.g., Bias, assumptions, reasons and interests in the research topic The Kaleidoscope research team were commissioned to conduct the interviews as part of their service offering	Methods—6
		Domain 2: Study design	
		Theoretical framework	
Methodological orientation and Theory	9	What methodological orientation was stated to underpin the study? E.g., grounded theory, discourse analysis, ethnography, phenomenology, content analysis Content analysis	Methods—6
		Participant selection	
Sampling	10	How were the participants selected? E.g., purposive, convenience, consecutive, snowball Purposive	Methods—5
Method of approach	11	How were the participants approached? E.g., face-to-face, telephone, mail, email Via a question as part of MS Forms survey; email	Methods—5
Sample size	12	How many participants were approached? There were a total of 37 participants over the five sessions. All participants were approached to participate in the interviews (via MS Forms survey and/or email). There were seven participants in the interviews	Methods—5 Results—6
Non-participation	13	How many people refused to participate or dropped out? Reasons? Participants of the programme were invited to participate in interviews. Participants were advised they were under no obligation to participate and could withdraw up until the point of final submission. All those who initially agreed to be interviewed completed an interview	Methods—6
		Setting	
Setting of data collection	14	Where was the data collected? E.g. home, clinic, workplace Via MS Teams with interview transcription onto Microsoft Word	Materials and Methods—5
Presence of non-participants	15	Was anyone else present besides the participants and researchers? No other individuals were present	Materials and Methods—6
Description of sample	16	What are the important characteristic of the sample? E.g., demographic data, date Interviews were conducted during May 2024. Of the seven interview participants, four were contractors, two were pharmacy managers and one was a SEL community pharmacy leader	Materials and Methods—5, 6 Results—9
		Data collection	
Interview guide	17	Were questions, prompts, guides provided by the authors? Was it pilot tested? Semi structured interviews were used. Face validation received	Materials and Methods—4, 5
Repeat interviews	18	Were repeat interviews carried out? If yes, how many? No	
Audio/visual recording	19	Did the research use audio or visual recording to collect the data? All interviews were audio recorded and transcribed	Materials and Methods—5
Field notes	20	Were field notes made during and/or/after the interview or focus group? No additional notes were made	Methods—5, 6
Duration	21	What was the duraction of the interviews or focus groups? The interviews lasted between 24 to 36 min	Results—9
Data saturation	22	Was data saturation discussed? All those who agreed to participate were included	Methods—4
Transcripts returned	23	Were transcripts returned to particpants for comments and/pr correction? yes	Methods—6
		Domain 3: analysis and findings	
		Data analysis	
Number of data coders	24	How many data coders coded the data? Transcripts were read by two members of the research team FR, RM)	Methods—6
Description of the coding tree	25	Did authors provide a description of the coding tree? Inductive content analysis was used	Methods—6
Derivation of themes	26	Were themes identified in advance or derived from the data? Inductive content analysis was used.	Methods—6
	**Item No**	**Guide Guides/Description**	**On Page No**
Software	27	What software, if applicable, was used to manage the data? Data was analysed manually	Methods—6
Participant checking	28	Did participants provide feedback on the findings? Yes—confirmation of interview transcriptions	Methods—6
		Reporting	
Questions presented	29	Were participant quotations presented to illustrate the themes/findings? Was each quotation identified? E.g., participant number Comments were supported with direct quotes from participants who were anonymised	Methods—6 Results—9–13
Data and findings consistent	30	Was there consistency between the data presented and the findings? Yes	Results—9–13
Clarity of major themes Clarity of minor themes	31 32	Were major themes clearly presented in the findings? Yes Is there a description of diverse cases or discussion of minor themes? No	Results—9–13
